# Radiation-Associated Sarcomatoid Breast Tumor 12 Years After Breast-Conserving Therapy: A Case Report From the Caribbean

**DOI:** 10.7759/cureus.117617

**Published:** 2026-10-07

**Authors:** Krystan S Hosein, Shawnessa A Chaitan, Ryan Richardson, Thane D Guerra, Shiva Sarwan, Dale Hassranah

**Affiliations:** 1 Department of General Surgery, Sangre Grande Hospital Campus, Sangre Grande, TTO

**Keywords:** breast-conserving surgery, complications of radiotherapy, post-radiation sarcoma, radiation-induced breast sarcoma, sarcomatoid carcinomas, secondary malignancy

## Abstract

Radiation-associated malignancies are rare but serious late complications of radiotherapy. Radiation-induced sarcoma is traditionally defined by Cahan's criteria, i.e., origin within a previously irradiated field, a latency period exceeding five years, development from previously normal tissue, and histological distinction from the primary malignancy. We report a rare radiation-associated sarcomatoid malignancy of the breast in a Caribbean patient. A 65-year-old woman presented with a rapidly enlarging breast mass 12 years after being treated with breast-conserving surgery, axillary clearance, adjuvant radiotherapy and endocrine therapy for stage I, hormone receptor-positive, invasive ductal carcinoma of the left breast in 2013. Imaging demonstrated a large, necrotic lesion with chest wall involvement and no distant metastases. Core biopsy revealed a high-grade sarcoma. Immunohistochemistry was largely negative for epithelial and mesenchymal markers, with a high proliferative index (Ki-67: 40%). The patient underwent radical mastectomy, and the final histopathological examination demonstrated a grade 3 sarcomatoid tumor with clear margins. Given its development within a previously irradiated breast after a 12-year latency, a radiation-associated malignancy was considered. Complete surgical resection with negative margins remains an important component of management of localized radiation-associated sarcomatoid malignancies. The role of adjuvant systemic therapy and re-irradiation remains unclear and should be individualized through multidisciplinary discussion. Radiation-associated malignancies, although rare, should be considered in patients presenting with new breast masses years after radiotherapy. Early recognition and prompt evaluation are essential to facilitate curative management. This case highlights the importance of long-term survival and multidisciplinary care in breast cancer survivors.

## Introduction

Radiation-induced sarcoma (RIS) is a rare, late complication of radiotherapy and has a cumulative incidence of approximately 0.3% at fifteen years after treatment, with breast cancer being the most common primary tumor [1,2]. According to Cahan's criteria, radiation-induced sarcoma must arise within a previously irradiated field, have a latency period of more than five years, develop from previously normal tissue, and be histologically distinct from the primary tumor [3]. Radiation-associated breast malignancies may present many years after treatment and can pose diagnostic challenges, particularly when sarcomatoid morphology is encountered. We report a rare case of a sarcomatoid tumor arising in a previously irradiated breast approximately 12 years after breast-conserving therapy.

## Case presentation

A 65-year-old woman with a past medical history of hypertension, diabetes mellitus, and left breast cancer was treated in 2013 with wide local excision and level two axillary clearance. Histopathological examination confirmed invasive ductal carcinoma, grade II, stage I (T1N0M0), which was estrogen receptor (ER)-positive, progesterone receptor (PR)-positive, and human epidermal growth factor receptor two (HER2)-negative. She subsequently received adjuvant radiotherapy; however, the dose, fractionation, and treatment field were not available in the records reviewed. She received tamoxifen for one year, followed by anastrozole for nine years. The patient had routine annual follow-up and imaging for 12 years thereafter. Her latest surveillance imaging in September 2024 showed no evidence of recurrence.

However, in April 2025, during routine oncological follow-up, she presented with a two-month history of a new, rapidly enlarging mass in the left breast. The patient denied any pain or nipple changes. Clinical examination revealed a firm, 6 x 6 cm, non-tender mass, extending from the left upper quadrant to the left lower quadrant of the left breast and fixed to the chest wall. The overlying skin showed no erythema, dimpling, or ulceration, and the axillary lymph nodes were not palpable. In May 2025, core (Tru-Cut) biopsy demonstrated a high-grade sarcoma with a “hint” of myoid differentiation (Figure 1).

**Figure 1:**
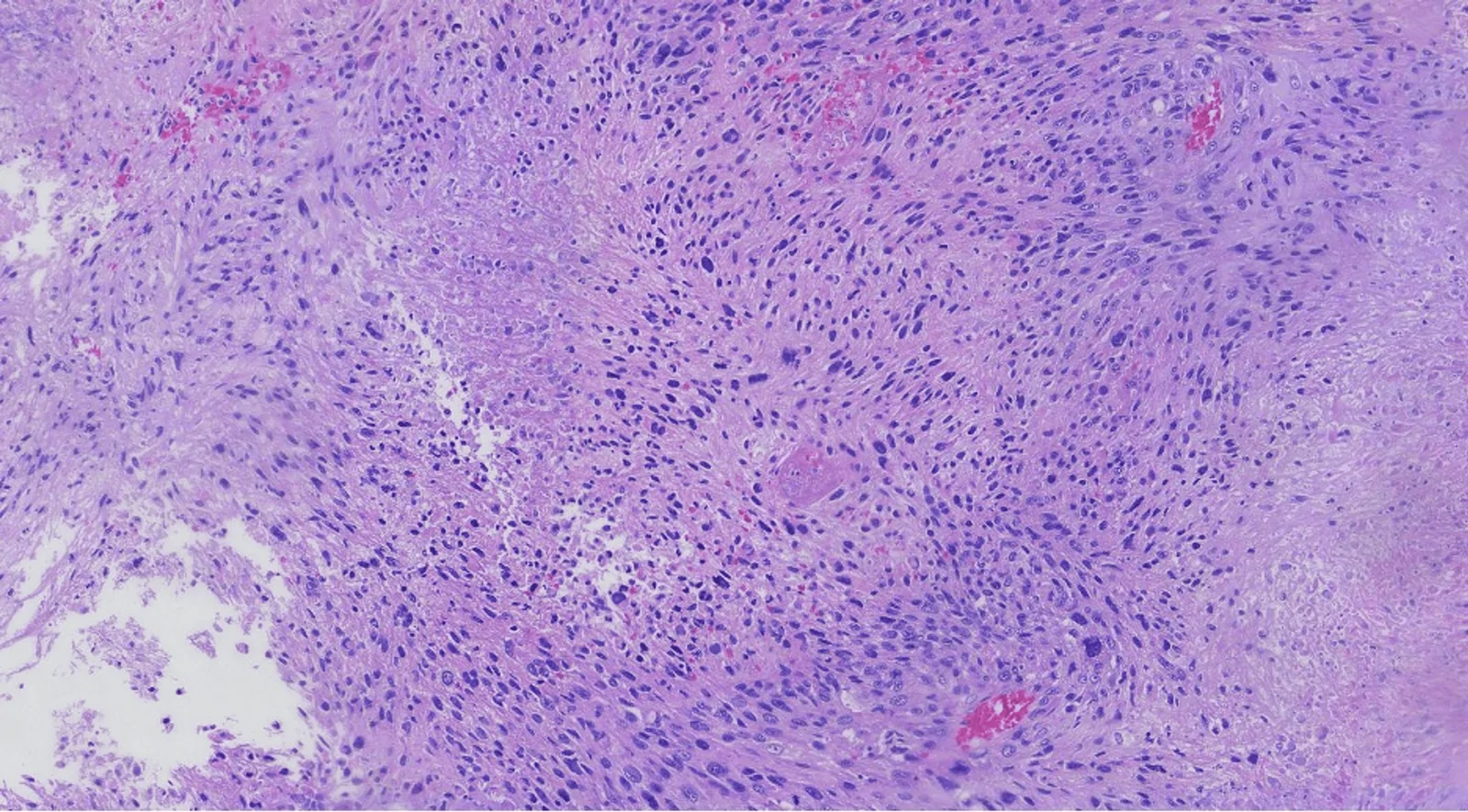
High-grade sarcoma on H&E staining, composed of cells with abundant cytoplasm and ovoid nuclei, arranged in sheets and vague fascicles with occasional multinucleated cells with numerous mitoses.

Immunohistochemical examination demonstrated predominantly negative cytokeratin staining with weak nonspecific reactivity, caldesmon (E89) focally positive (Figure 2), BRG1 (EPNCIR111A) retained, AE1/AE3 mostly negative with only focal, weak, nonspecific staining, and Ki-67 approximately 40%. The tumor was ER-negative, PR-positive, HER2-negative, and had a Ki-67 proliferation index of 40%. The overall immunohistochemistry and molecular analysis are outlined in Table 1. Differentials included sarcomatous transformation of the previous tumor or de novo sarcoma; however, sample bias could not be excluded.

**Figure 2:**
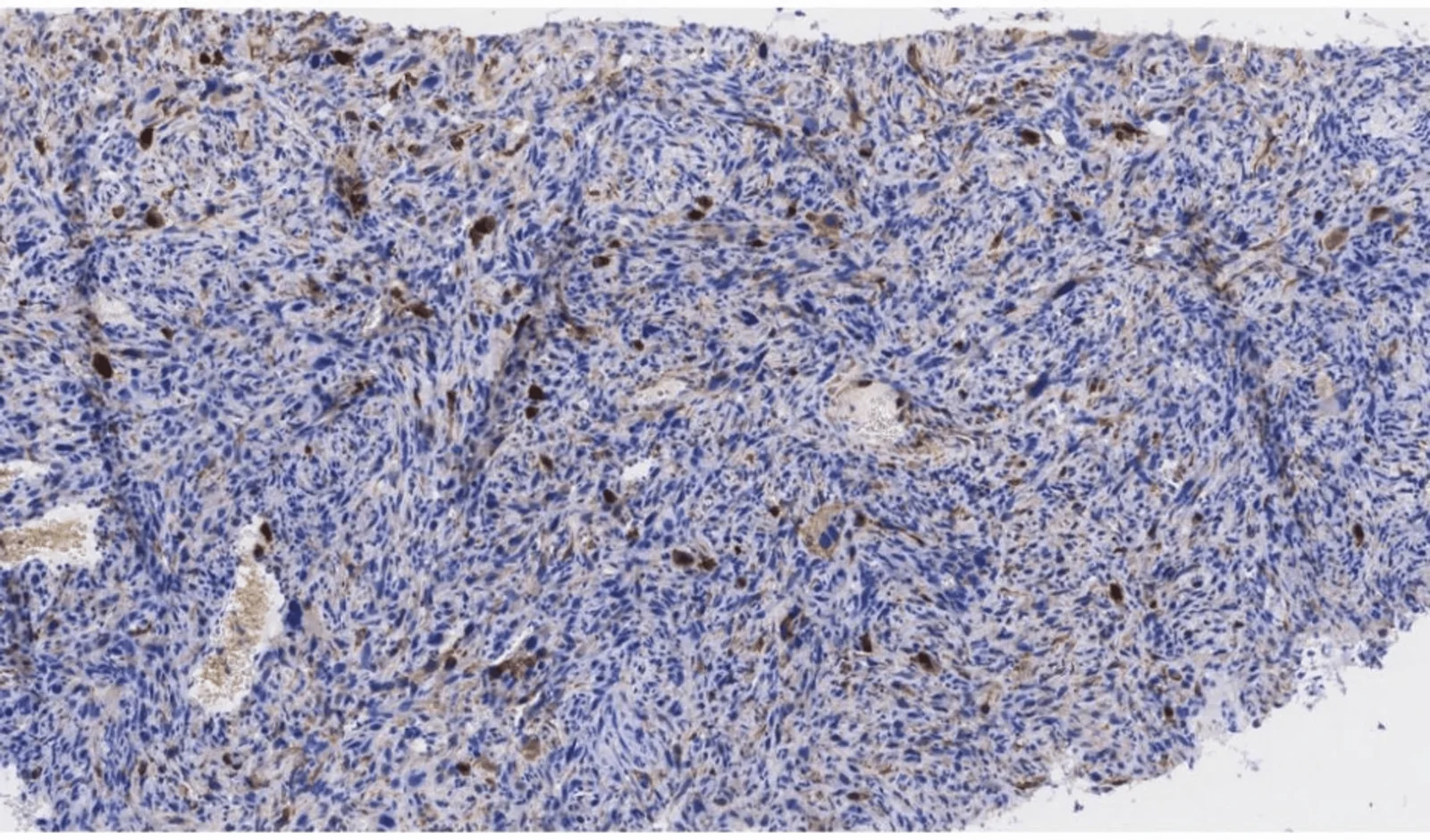
Immunohistochemical staining demonstrating focally H-caldesmon positivity in the high-grade sarcoma.

**Table 1:** Immunohistochemical profile of the Tru-Cut biopsy specimen. ALK: anaplastic lymphoma kinase; SMA: smooth muscle actin; ER: estrogen receptor; PR: progesterone receptor; HER2: human epidermal growth factor receptor two.

Marker	Expression
AE1/AE3	Mostly negative with only focal, weak, nonspecific staining
ER	Negative (0/8)
PR	2+ (5/8), H-score 30 (10% strong staining)
HER2	Negative (0)
Keratin OSCAR	Negative
p63/CK5 (4A4/XM26)	Negative
S100 (EP32) (Polyclonal)	Negative
SMA (1A4)	Negative
Desmin (D33)	Negative
SOX10 (BC34)	Negative
Caldesmon (E89)	Focally positive
ALK (D5F3)	Negative
BRG1 (EPNCIR111A)	Retained
CD34 (QBEND/10)	Negative

In June 2025, contrast-enhanced computed tomography (CECT) of the chest, abdomen, and pelvis (Figures 3, 4) demonstrated a centrally necrotic left breast mass measuring 6.1 cm x 5.6 cm x 5.7cm in the anteroposterior, transverse, and craniocaudal planes, respectively. The lesion was associated with apparent chest wall involvement and overlying skin thickening. No axillary, supraclavicular, or internal mammary lymphadenopathy or distant metastatic disease was identified. Clinical and radiological findings were consistent with stage cT4N0M0 disease. The case was discussed at a multidisciplinary team meeting, consisting of specialists in general surgery, oncology, and radiology, and the decision was made to perform a mastectomy.

**Figure 3:**
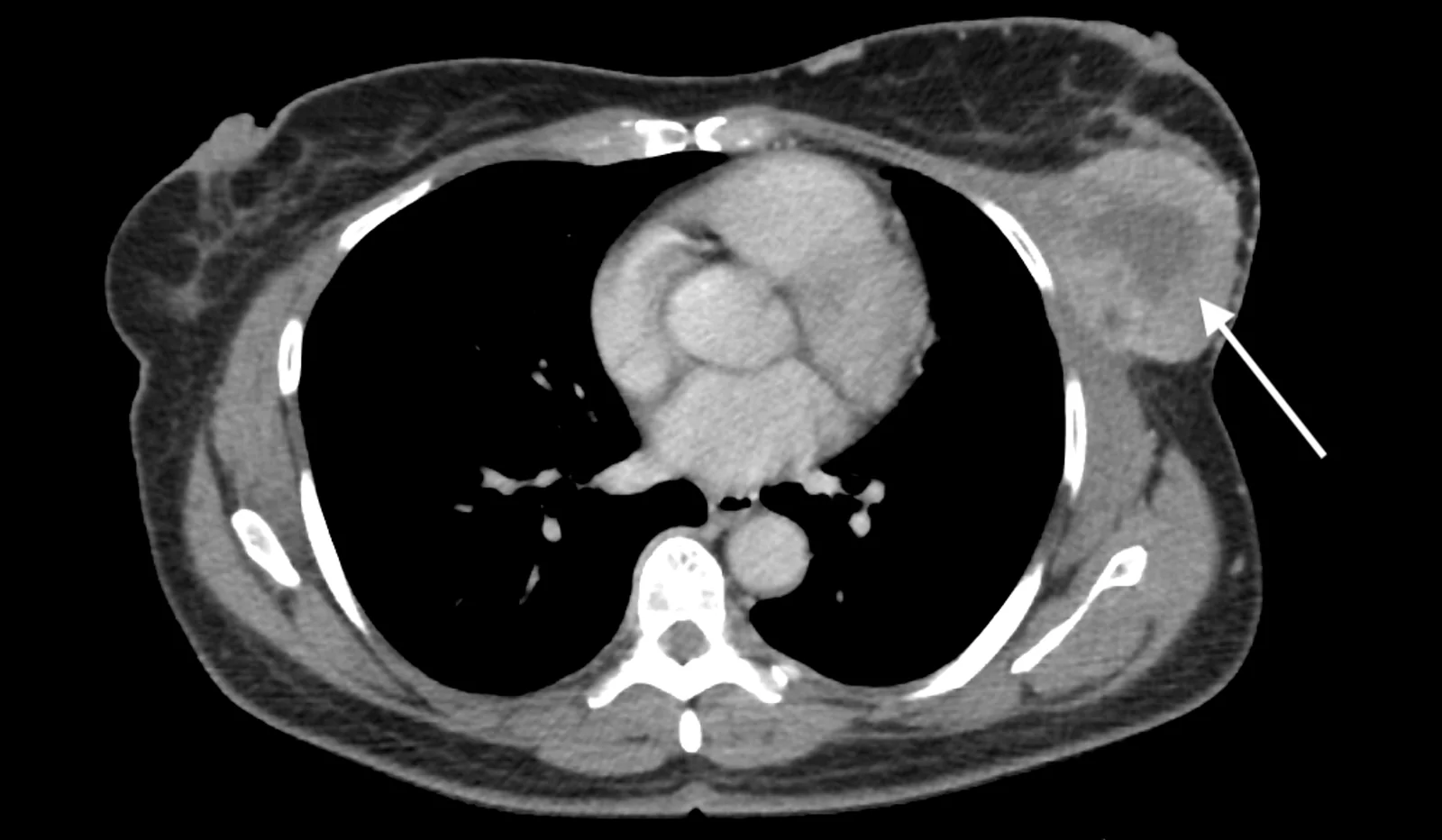
Axial contrast-enhanced CT demonstrating a centrally necrotic mass in the left breast with apparent chest wall involvement and overlying skin thickening (white arrow).

**Figure 4:**
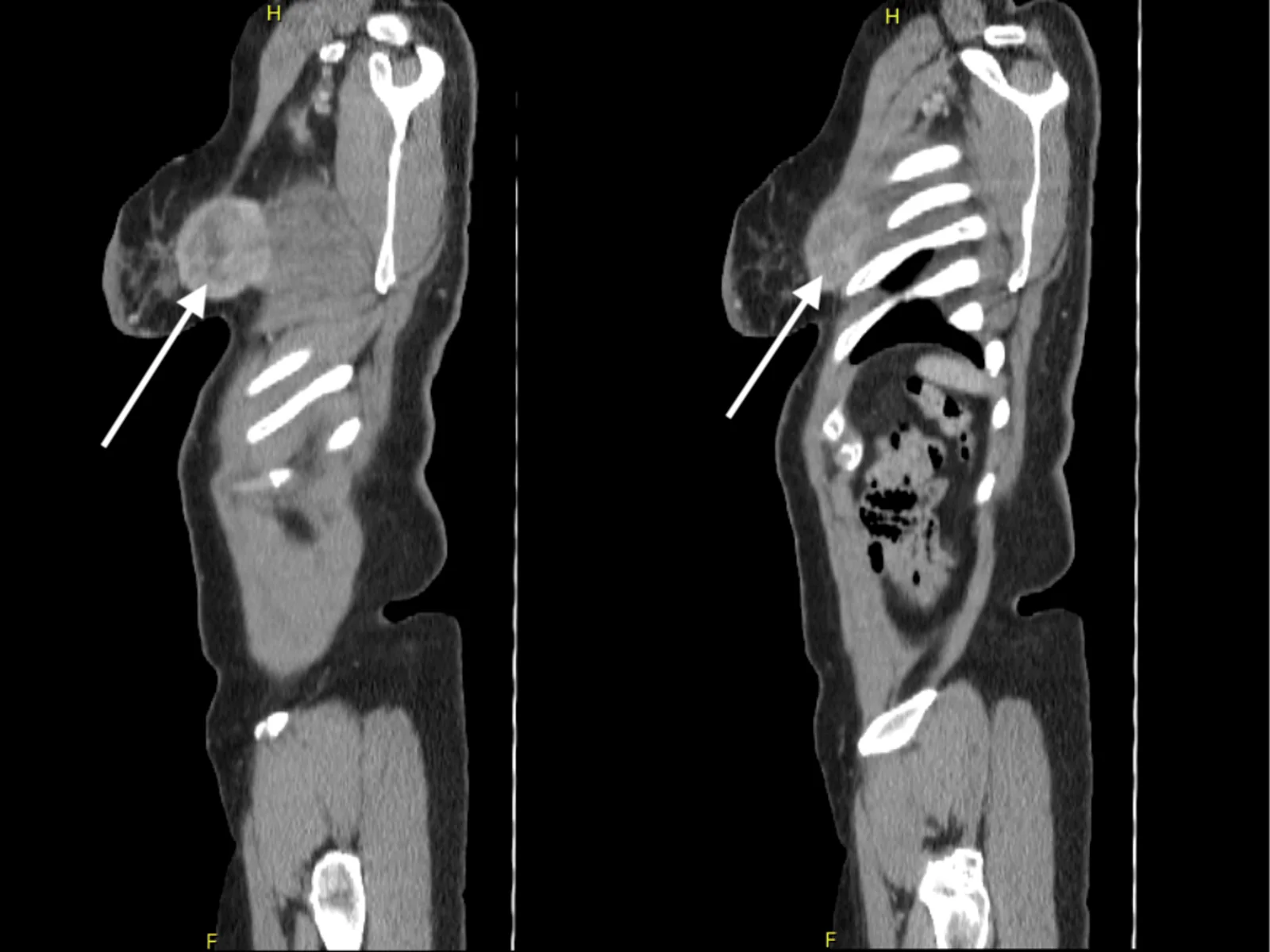
Sagittal slices of CECT demonstrating a centrally necrotic mass in the left breast with apparent chest wall involvement and overlying skin thickening (white arrows). CECT: contrast-enhanced computed tomography.

In August 2025, a left radical mastectomy was performed to remove a large multi-lobulated tumor, which extended laterally into the axillary tail. The patient was noted to have a “frozen axilla,” i.e., dense fibrotic change, which precluded further nodal dissection. Histopathological examination of the resection specimen demonstrated a grade III sarcomatoid tumor measuring 7.0 x 9.5 x 7.1 cm, with no evidence of lymphovascular or perineural invasion. The tumor was located 70 mm from the medial margin, 10 mm from the lateral margin, 2 mm from the anterior (skin) margin, 1 mm from the posterior margin, 11 mm from the superior margin, and 10 mm from the inferior margin. Microscopy demonstrated a pleomorphic sarcomatoid tumor composed of sheets and fascicles of cells with abundant, eosinophilic cytoplasm and markedly enlarged nuclei (Figure 5).

**Figure 5:**
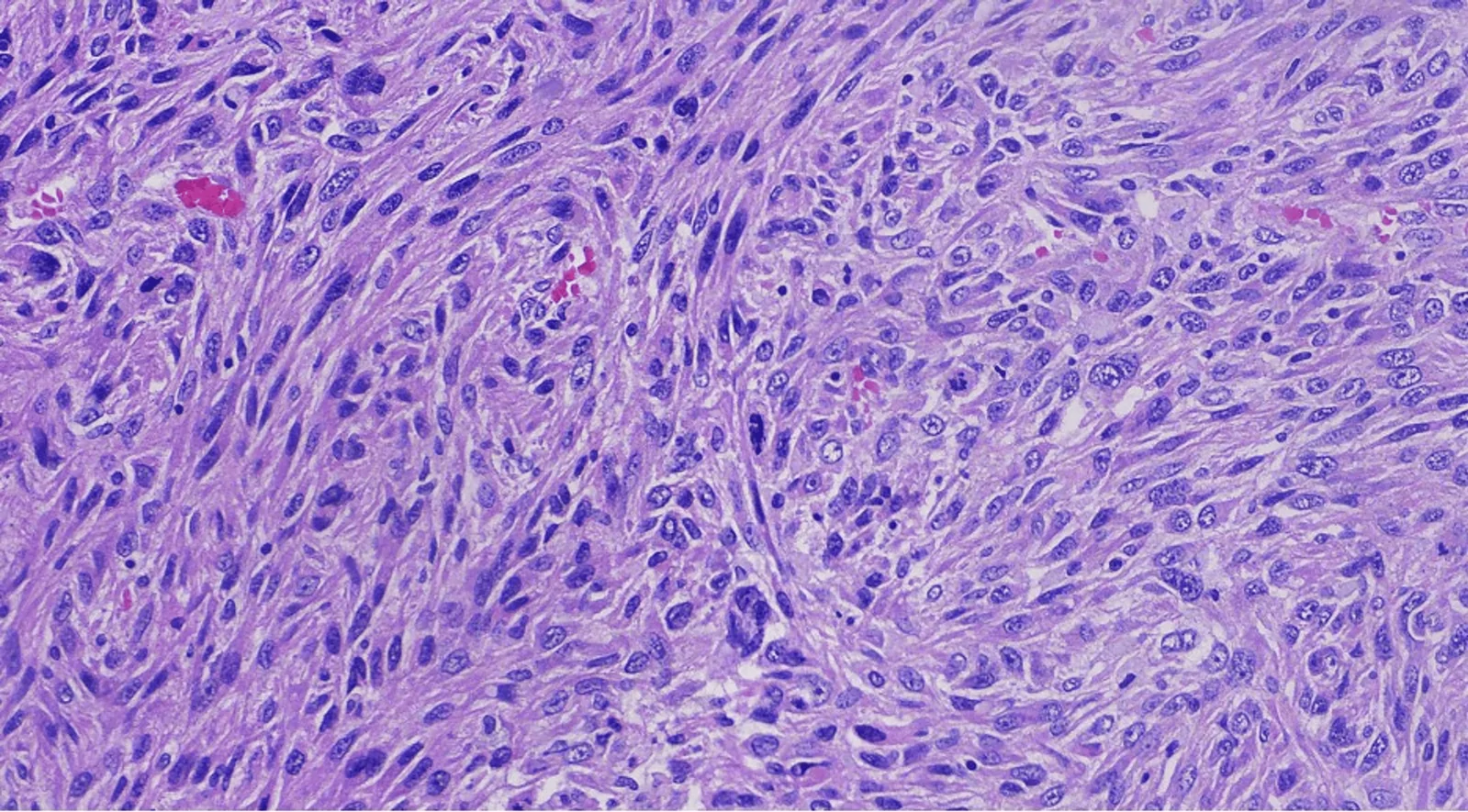
Histological examination of the resection specimen demonstrating a pleomorphic sarcomatoid tumor composed of sheets and fascicles of cells, with abundant eosinophilic cytoplasm and markedly enlarge appearing nuclei.

With these pathological findings and no evidence of metastatic spread on thoracic-abdominal-pelvic CT scan, the sarcomatoid tumor was therefore classified as pT2N0M0 (stage IIIA, the American Joint Committee on Cancer (AJCC) 8th Edition Cancer Staging Manual, soft tissue sarcomas) [4]. Further pathological subtyping of the resection specimen was available but was not performed. Consequently, although the clinical history and morphology supported a radiation-associated sarcomatoid malignancy, definitive classification as a specific sarcoma subtype was not established. The patient refused adjuvant chemotherapy and at 10 months following surgery, the patient remained clinically and radiologically free of recurrent or metastatic disease.

## Discussion

Breast-conserving surgery (BCS) followed by adjuvant radiotherapy is the standard of care for the management of early-stage breast cancer. Adjuvant whole-breast radiotherapy significantly reduces local recurrence and improves survival; however, it is associated with potential long-term complications [5,6].

Radiation-induced sarcoma of the breast is a rare but serious late complication of radiotherapy, with a reported incidence ranging from approximately 0.03% to 0.3% at 15 years. Despite its low incidence, RIS occurs more frequently following breast cancer treatment compared to other primary solid-tumor cancers [7].

The clinical characteristics of this case are suggestive of a radiation-associated malignancy. The tumor developed in the previously irradiated left breast approximately 12 years after completion of radiotherapy, exceeding the latency period traditionally described in Cahan's criteria. The original malignancy was a grade 2 invasive ductal carcinoma, whereas the subsequent lesions demonstrated a high-grade sarcomatoid morphology; however, the absence of definitive sarcoma subtyping limits strict application of the classical Cahan criteria.

Multiple factors guided the decision to proceed with a mastectomy, including the large tumor size, apparent chest wall invasion, extensive local disease, and high-grade sarcomatoid histology. The goal was to achieve complete oncological resection with negative margins. In such cases, breast-conserving surgery is generally not feasible or oncologically safe due to the need for wide surgical margins and the aggressive local behavior of the tumor. Complete surgical excision with negative margins is the most important factor for local control and survival and remains the cornerstone of treatment for localized RIS [8,9]. In the present case, mastectomy achieved clear surgical margins.

The role of adjuvant therapeutic options, including systemic therapy and re-irradiation, remains controversial, and standardized guidelines are not well established [10,11]. These tumors have often demonstrated limited response to conventional chemotherapeutic agents; however, systemic therapy may be considered in selected cases with high-grade or metastatic disease, but there is no standardized regimen proven to improve survival outcomes [12,13]. Re-irradiation is often avoided due to the high risk of toxicities such as soft tissue necrosis, rib fracture, and pneumonitis [14].

In this case, complete surgical resection was the sole treatment modality pursued with the aim of achieving optimal local control. Given the rarity of RIS and the lack of large prospective studies, management is often individualized and guided by multidisciplinary team discussion, considering patient-specific factors such as prior treatment exposure, performance status, and disease extent [11,15].

This case underscores the importance of recognizing radiation-induced malignancies as a potential late complication of breast-conserving therapy. Although radiotherapy plays a crucial role in improving local control and survival in breast cancer, its long-term adverse effects must be acknowledged. Radiation-induced malignancies may develop many years after treatment, sometimes beyond a decade and frequently present with aggressive clinical features [8]. Early detection is therefore essential for improving outcomes. Routine follow-up should include careful clinical examination and imaging where appropriate, and clinicians should maintain a high index of suspicion where patients present with rapidly enlarging, firm, or painful masses in a previously irradiated field. Biopsy and histologic assessment of any new breast lumps ensure early diagnosis and allow for potentially curative surgical resection [16,17].

Moreover, this case highlights the need for thorough patient pre-radiotherapy counseling. This ensures that patients are aware of the established therapeutic benefits and recognized short- and long-term adverse effects, including the rare risk of secondary malignancy. A multidisciplinary approach involving surgeons, oncologists, radiologists, and pathologists is critical for timely diagnosis and management of radiation-induced sarcoma, ultimately contributing to better long-term outcomes and improved quality of life for breast cancer survivors.

## Conclusions

Radiation-associated malignancies are a rare but important late complication of breast-conserving therapy. This case demonstrated the diagnostic complexity of a high-grade sarcomatoid tumor in a Caribbean patient. It highlights the importance of pre-radiation counseling as well as long-term follow-up in breast cancer survivors. Although radiotherapy remains an essential component of breast-conserving treatment, clinicians should remain vigilant for new or atypical lesions within previously irradiated fields. Early recognition and prompt surgical intervention offer the best chance for disease control, supported by continued patient education and multidisciplinary follow-up to improve outcomes and quality of life.
